# Interplay between maternal *Slc6a4* mutation and prenatal stress: a possible mechanism for autistic behavior development

**DOI:** 10.1038/s41598-017-07405-3

**Published:** 2017-08-18

**Authors:** Calvin P. Sjaarda, Patrick Hecht, Amy J. M. McNaughton, Audrina Zhou, Melissa L. Hudson, Matt J. Will, Garth Smith, Muhammad Ayub, Ping Liang, Nansheng Chen, David Beversdorf, Xudong Liu

**Affiliations:** 1 Department of Psychiatry, Queen’s University, Kingston, Ontario Canada; 2 Queen’s Genomics Lab at Ongwanada (QGLO), Ongwanada Resource Center, Kingston, Ontario Canada; 3 Interdisciplinary Neuroscience Program, University of Missouri, Columbia, Missouri USA; 4 Psychological Sciences and Bond Life Sciences Center, University of Missouri, Columbia, Missouri USA; 5 Department of Pediatrics, Queen’s University, Kingston, Ontario Canada; 6 Child Development Centre, Hotel Dieu Hospital, Kingston, Ontario Canada; 7 Department of Biological Sciences, Brock University, St. Catharines, Ontario Canada; 8 Department of Molecular Biology and Biochemistry, Simon Fraser University, Burnaby, British Columbia Canada; 9 Departments of Radiology, Neurology, and Psychological Sciences, and the Thompson Center for Autism and Neurodevelopmental Disorders, and William and Nancy Thompson Endowed Chair in Radiology, University of Missouri, Columbia, Missouri USA

## Abstract

The low activity allele of the maternal polymorphism, 5HTTLPR, in the serotonin transporter, *SLC6A4*, coupled with prenatal stress is reported to increase the risk for children to develop autism spectrum disorder (ASD). Similarly, maternal *Slc6a4* knock-out and prenatal stress in rodents results in offspring demonstrating ASD-like characteristics. The present study uses an integrative genomics approach to explore mechanistic changes in early brain development in mouse embryos exposed to this maternal gene-environment phenomenon. Restraint stress was applied to pregnant *Slc6a4* ^+/+^ and *Slc6a4* ^+/−^ mice and post-stress embryonic brains were assessed for whole genome level profiling of methylome, transcriptome and miRNA using Next Generation Sequencing. Embryos of stressed *Slc6a4* ^+/+^ dams exhibited significantly altered methylation profiles and differential expression of 157 miRNAs and 1009 genes affecting neuron development and cellular adhesion pathways, which may function as a coping mechanism to prenatal stress. In striking contrast, the response of embryos of stressed *Slc6a4* ^+/−^ dams was found to be attenuated, shown by significantly reduced numbers of differentially expressed genes (458) and miRNA (0) and genome hypermethylation. This attenuated response may pose increased risks on typical brain development resulting in development of ASD-like characteristics in offspring of mothers with deficits in serotonin related pathways during stressful pregnancies.

## Introduction

Autism Spectrum Disorder (ASD) is a complex neurodevelopmental condition characterised by deficits in two core domains: impairments in social communication and restricted and repetitive behaviors^1^. Although early reports on the prevalence of autism described a severe condition affecting 4.5 cases per 10,000 children in the 1960s^2^, recent studies report a spectrum disorder affecting 1 in 68 children^3^. There is no consensus in the scientific community about the main cause for this increase, while much is ascribed to new administrative classifications, policy and practice changes and increased awareness^4, 5^, it is also commonly agreed that environmental and genetic factors play important roles^6^. ASD has a complex etiology with large genetic and significant environmental components with prenatal maternal stress^7^ having been shown to be an important non-genetic factor associated with development of ASD.

Serotonin (5-HT) functions as a neurotransmitter in the central nervous system (CNS) where it is involved in a range of behaviors and psychological processes including mood, appetite, anxiety, social interactions, and hormone release. Studies have reported increased whole blood 5-HT levels in individuals with ASD^8–10^ causing a negative feedback response in the CNS, resulting in a 5-HT deficit in certain target areas in the brain^11–13^. This has been demonstrated by positron emission tomography scans showing reduced 5-HT synthesis and changes in receptor density in the cortex and thalamus of autistic individuals^14–16^. A polymorphism investigated extensively is an allelic variant, 5-HTTLPR, within the promoter region of the *SLC6A4* gene which codes for the serotonin transporter (SERT) protein. The 5-HTTLPR is a functional polymorphism located ~1 kb upstream of the transcription start site and consists of a long (L), higher activity allele and a short (S), lower activity allele^17^. Cells with the L/L genotypes of 5-HTTLPR have 1.9–2.2 times higher 5-HT uptake than cells with one or two copies of the S allele^18^. Individuals carrying an S allele demonstrate greater amygdalar neuronal activity in response to negative environmental cues and fearful stimuli^19^ and are more susceptible to anxiety and stress^20^. Multiple studies have reported association of 5-HTTLPR with individuals with ASD^21–24^.

Maternal stress causing adverse psychopathological outcomes in the offspring has been reported for many psychological stressors^25–27^. Our early studies on humans suggest that prenatal maternal stress can cause cognitive, behavioral, physical and emotional problems in the offspring including ASD^7^. Many factors can modulate the impact of prenatal maternal stress on development of ASD including timing of stress^7, 28^, coping mechanism of the mother^29, 30^, and genetic susceptibility of the mother and embryo^30^.

Our more recent study reported that the maternal 5-HTTLPR polymorphism in *SLC6A4*, coupled with prenatal stress, may significantly affect the risk for offspring to develop ASD^31^. Specifically, mothers of children with ASD and who carry the stress-susceptible, short allele of the 5-HTTLPR locus had higher incidences and severity of stress during their affected pregnancies. This conclusion was based on two observations: mothers carrying the short allele of the 5-HTTLPR loci reported more stressors compared to mothers homozygous for the long allele during their affected pregnancy, and the same mothers reported no significant stress exposure during their pregnancies resulting in typically developing children, regardless of maternal genotype^31^. These results suggest that the relationship between this allele and history of stress exposure in ASD is specific to ASD and is not simply due to increased recall of stressors during pregnancy in the presence of this allele. This interaction has been modeled in rodents, since *Slc6a4* ^+/−^ rodents under adverse environmental conditions develop behavioral changes including acoustic startle, learned helplessness^32^, and increased fear, anxiety and depression that are consistent with humans carrying a short allele of the 5-HTTLPR locus^18, 33–35^. Evidence in mice models indicate that when dams with a heterozygous knockout in the *Slc6a4* gene are exposed to stress, their offspring have increased risk to demonstrate increased anxiety and ASD-like characteristics including decreased social interaction and social interest^36^. In addition, reports have shown that the homozygous or heterozygous knockout in the *Slc6a4* and/or prenatal stress in the dam affects the epigenetic signatures and transcriptome profiles in newborn and adult offspring^37–39^. In short, mothers with aberrant serotonin function (via reduced *SERT* expression in humans and *Slc6a4* gene knockout in mice) respond differently to stress, the differential stress response is passed to the developing embryo, which increases their risk of developing ASD and ASD-like characteristics. In this study, using integrative genomics approaches, we describe the transcriptome, miRNA and methylome response of developing embryos to maternal cues stemming from the interplay between maternal genotype and prenatal stress.

## Methods and Materials

### Animal model

*Slc6a4* ^−/−^ male mice were bred with wild-type (WT: *Slc6a4* ^+/+^) female mice on a C57BL/6J background (Jackson Laboratories, Bar Harbor, ME). These mice contain a neomycin selection cassette that replaced a DNA segment containing exon 2 in the *Slc6a4* gene. Mice with a heterozygous knockout (KO) of the *Slc6a4* gene have a 50% reduction in SERT density, but similar serotonin transport when compared with WT mice, while homozygous KO mice have no SERT and no serotonin transport^33^. The behaviors of mice with reduced or absent *Slc6a4* expression (homozygous and heterozygous KO) has been reviewed in detail by Murphy *et al*.; but briefly include: an increase in anxiety, depression, startle response, and response to stress, as well as reduced learning capacity in aggression tests which may be related to deficits in other social interactions tasks^32^. WT female offspring underwent another round of breeding with male *Slc6a4* ^+/−^ mice to generate the experimental dams and sires used in this study. Experimental dams (*Slc6a4* ^+/+^ and *Slc6a4* ^+/−^) were housed in pairs of two and bred with WT males and identification of a vaginal plug was marked embryonic day 0.5 (E0.5). Three pregnant mice were randomly selected from each genotype for the stress group and subjected to acute restraint stress on E12.5, as described in the Supplementary Information. All experimental procedures were approved by and conducted in accordance with the University of Missouri Institutional Animal Care and Use Committee and the University of Missouri Internal Review.

No statistical methods were used to pre-determine sample sizes, but we followed best practices in RNA-seq which stipulates three biological replicates as the minimum for inferential analysis^40^. Placenta and embryonic brain tissue was collected from E13.5 mice and used as a source of genomic DNA and total RNA. We collected tissue from 3 placentas and 3 embryos for each experimental maternal condition (WT control, SERT control, WT stress, SERT stress) for a total of 12 placenta and 12 embryo brain samples (Table 1). Dams and embryos were genotyped for the *Slc6a4* allele with the EZ Fast Tissue/Tail PCR Genotyping Kit as per manufacturer’s instructions (EZ BioResearch). Tail clippings were collected from mice at the time of weaning and from embryonic tissue at the time of tissue collection. The following primers were used for the PCR reaction: Forward: 5′-AATGGTGAGGAGTGGTGGAG-3′; Wild-type Reverse: 5′-CCTAGATACCAGGCCCACAA-3′; Knockout Reverse: 5′-GCCAGAGGCCACTTGTGTAG-3′. Embryos had a 50% chance of inheriting the knockout *Slc6a4* from their mother; to avoid embryo genotype from confounding the effects of the interaction between maternal genotype and environmental stress, *Slc6a4* ^+/−^ embryos were removed from the study (sample E72L1: the first embryo in the left uterine horn (L1) obtained from dam 72 (E72) was removed). To aid in differentiating between groups of samples in this manuscript, embryos growing within *Slc6a4* ^+/+^ dams are referred to as WT embryos and within *Slc6a4* ^+/−^ dams are called SERT embryos even though all embryos were determined to be *Slc6a4* ^+/+^.

**Table 1:** Sample summary of embryo brain tissue.

Sample	*Slc6a4* genotype of mother	Treatment	*Slc6a4* genotype of embryo	Sex of embryo
E74L1	+/+	Control	+/+	F
E59R5	+/+	Stress	+/+	F
E69R7	+/−	Stress	+/+	M
E77L1	+/+	Control	+/+	F
E72L1	+/−	Control	+/−	M
E67R9	+/+	Stress	+/+	M
E75L1	+/−	Control	+/+	F
E81R9	+/−	Stress	+/+	F
E23L1	+/+	Control	+/+	M
E48R5	+/−	Control	+/+	F
E18R2	+/+	Stress	+/+	M
E83L6	+/−	Stress	+/+	F

I. Sample identifier: E74L1 refers to the first embryo in the left uterine horn (L1) obtained from dam 74 (E74) II. E72L1: removed from study because embryo was heterozygous for *Slc6a4*. III. E23L1: was removed from methylome analysis because library was not enriched for methylated DNA.

### Library preparation and sequencing

Nucleic acids were collected from placenta and embryo brains and used as the template for methylome, transcriptome and miRNA library construction, followed by sequencing on the Ion Proton System (Thermofisher Scientific, Carlsbad, CA). Tissue collection, nucleic acid isolation, library preparation and sequencing are described in the Supplementary Information. For each sample, we generated 19.40 ± 1.03 million mapped reads for methylome libraries, 18.86 ± 2.10 million reads for transcriptome libraries and 4.77 ± 0.13 million reads for miRNA libraries.

### Bioinformatic analysis

DNA methylation was assessed using R-based pipelines MEDIPS^41^ and MethylAction^42^ with parameters described in the Supplementary Information. Differentially methylated regions (DMRs) called by both pipelines were used in the downstream analysis. One sample with low enrichment of methylated DNA was removed from further methylome analysis (Table 1). Genomic features and context were resolved using the R package Goldmine^43^ using default arguments. The global methylation index was determined as area under the curve using the trapezoidal approximation.

Transcriptome and miRNA raw reads were imported into Partek Flow version 5.0.16.1113 and analyzed with the WT pipeline for Ion Torrent pipeline and microRNA Bowtie pipeline respectively. Differentially expressed genes (DEGs) and miRNA were selected by p-value ≤ 0.05, fold change >1.5 or <−1.5 and average coverage of ≥15 normalized reads based on assessment of ERCC RNA Spike-In Control Mixes (Supplementary Fig. S1). A subset of DEGs was validated by qPCR as described in the Supplementary Information and Supplementary Table S1. Gene ontology (GO) pathway analyses were carried out using WEB-based GEne SeT AnaLysis Toolkit (http://bioinfo.vanderbilt.edu/webgestalt/login.php/)^44, 45^ using the mouse genome as the reference, significance p-value <0.05 with the Benjamini & Hochberg multiple test adjustment. TargetScan7.0 was used to discover genes targeted by differentially expressed microRNA (DE miRNA) (http://www.targetscan.org/mmu_71/)^46^. We downloaded the Summary Counts, default predictions files and extracted the 157 mouse miRNAs that were differentially expressed in our study with their potential mRNA targets. The number of targets was reduced by removal of all mRNA that were not differentially expressed in our study. Interactions between miRNA and their targeted genes were networked using Cytoscape^47^.

### Statistics

Statistical significance was calculated using IBM SPSS Statistics Version 24 software. Genome methylation index was compared using one-way ANOVA followed by Tukey post-hoc test. A test for homogeneity of variances did not show any significant variance between groups (df1 = 3, df2 = 6, p = 0.170). Correlation of gene expression measured by qPCR and RNA-seq was determined using Pearson correlation coefficient. Data are indicated as mean ± standard deviation. Data distribution was assumed to be normal, but this was not formally tested.

### Data availability

Embryo brain sample metadata is provided in Table 1. DMRs are listed in Supplementary Table S2A–C, DEGs are listed in Supplementary Table S3A–C and DE miRNAs are listed in Supplementary Table S4A,B.

## Results

To aid in differentiating between groups of samples in this manuscript, embryos growing within *Slc6a4* ^+/+^ dams are referred to as WT embryos and within *Slc6a4* ^+/−^ dams are called SERT embryos even though all embryos were determined to be *Slc6a4* ^+/+^.

### Methylome profiling of embryo brain tissues

**Figure 1:**
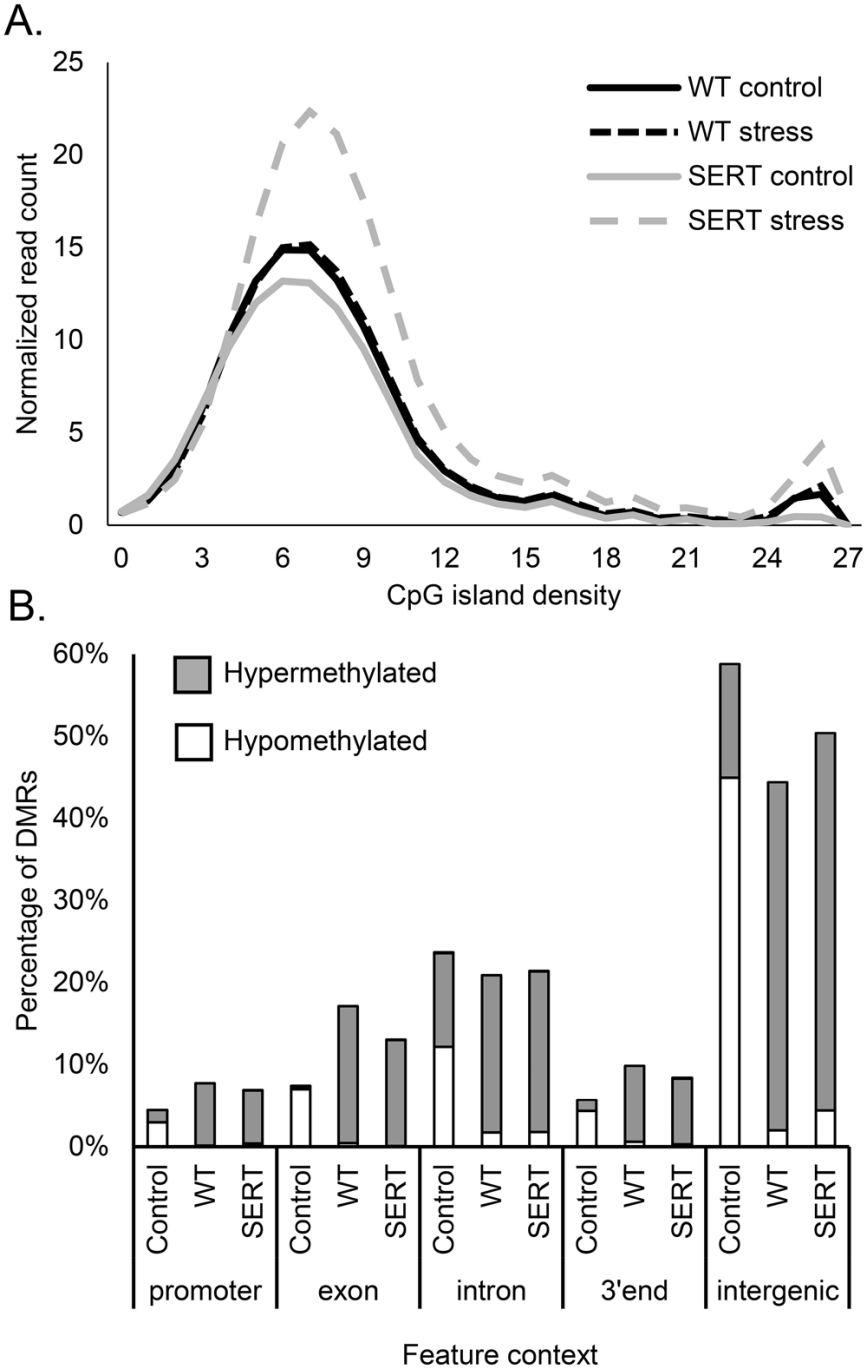
Embryo’s methylation profile is impacted by maternal effect. (**A**) The global methylation index quantifies genome wide methylation level for areas with similar CpG island density. SERT embryos exposed to prenatal stress display significant increase in genome methylation (one-way ANOVA F (3,6) = 5.92, p = 0.032 followed by Tukey post-hoc test p = 0.038). (**B**) Gene annotation links the genomic ranges containing differentially methylated regions (DMRs) to the gene model contexts. Control: the difference between maternal *Slc6a4* ^+/−^ (SERT) embryos and wild-type (WT) embryos without stress, WT: the effect of stress on the WT embryos, and SERT: the effect of stress on SERT embryos.

Without stress, SERT control embryos displayed 2,398 DMRs (77.5% hypomethylated) compared with the WT embryos (Supplementary Table S2A), though the overall global methylation index was not significantly different between the two groups (WT control area under the curve (AUC) = 116.47 ± 4.57, n = 2; SERT control AUC = 102.41 ± 25.48, n = 2) (Fig. 1A). When mothers were subjected to the stress paradigm, WT embryos displayed 844 DMRs (95.3% hypermethylated) compared to embryos without stress (Supplementary Table S2B) but no significant change in the global methylation index (WT stress AUC = 118.41 ± 22.41, n = 3) was observed, while SERT embryos displayed significantly increased global methylation (SERT stress AUC = 170.54 ± 19.55, n = 3) (Fig. 1A) due to the presence of 13,512 DMRs (93.7% hypermethylated) (Supplementary Table S2C).

Annotation of DMRs based on its genomic context illustrated that most DMRs in the SERT control groups map to intergenic (58.81%) and intronic (23.68%) regions of the genome when compared with the WT control group (Fig. 1B). DMRs found in embryos under stress were present in higher proportions in the promoter (WT 7.75%, SERT 6.89%), exon (WT 17.14%, SERT 13.00%) and 3′ end (WT 9.86, SERT 8.37) when compared with their respective embryos without stress (Fig. 1B).

### Transcriptome profiling

**Figure 2:**
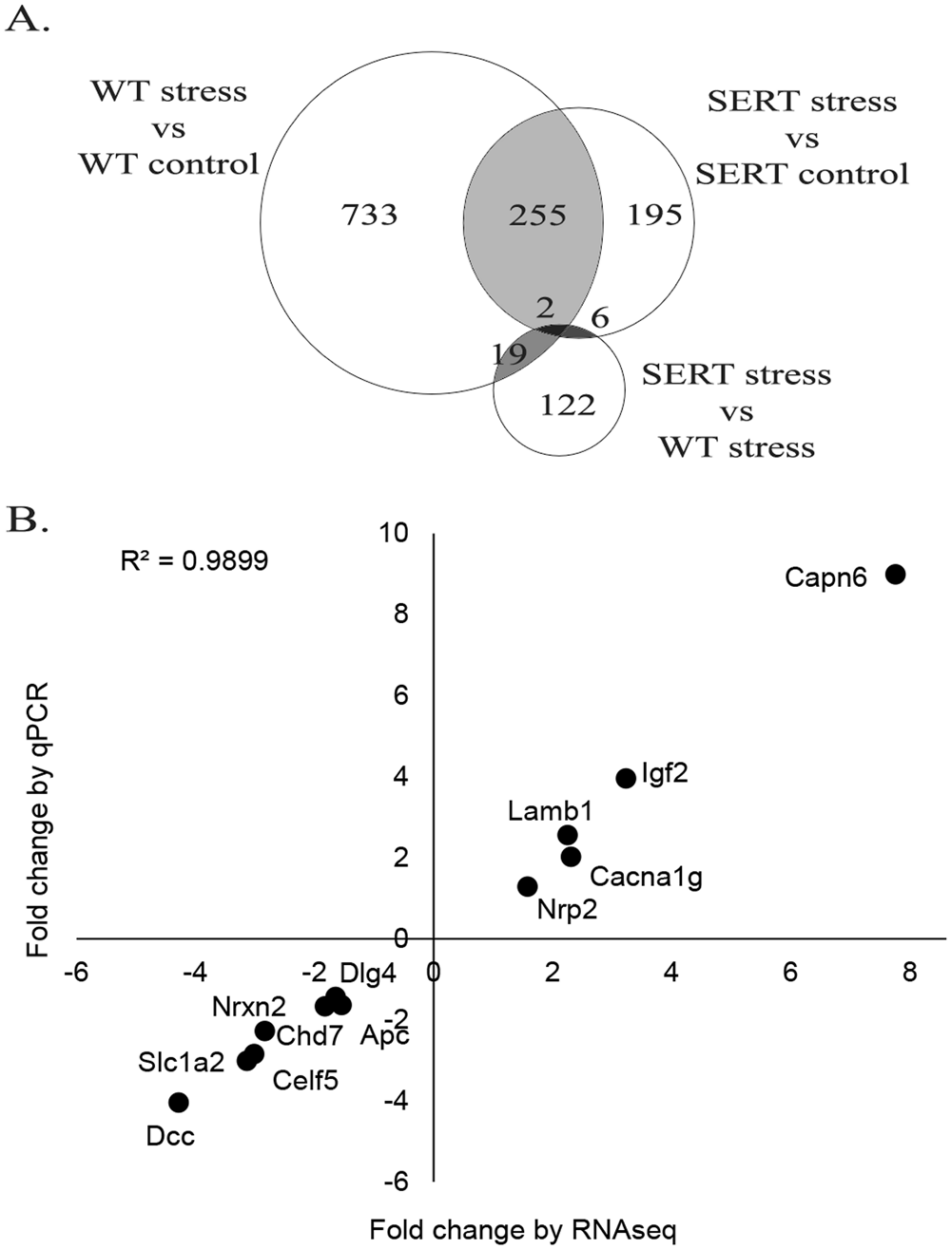
Maternal genotype and prenatal stress affect the transcriptome of developing embryos. (**A**) Venn diagram showing overlap between differentially expressed genes (DEGs) in wild-type (WT) and maternal *Slc6a4* ^+/−^ (SERT) embryos in response to stress. (**B**) Validation of subset of DEGs identified by RNA-seq using qPCR correlated with fold change from RNA-seq data (Pearson R = 0.995, p < 0.001, R^2^ = 0.9899).

Whole transcriptome profiling of the embryo’s brains revealed there were zero DEGs in the comparison between the SERT and WT control embryos. When mothers were subjected to the stress paradigm, WT embryos responded by differentially expression of 1,009 genes (61.5% upregulated) (Supplementary Table S3A) and the SERT embryos differentially expressed 458 genes (53.1% upregulated) (Supplementary Table S3B). There were 257 DEGs shared in the WT stress and SERT stress embryos (Fig. 2A). The genotype x environment interaction (SERT stress versus WT stress) identified 149 genes that were differentially expressed between the two groups (Supplementary Table S3C). RNA-seq expression levels were validated by quantitative real-time PCR (qPCR) for 12 DEGs reflecting various levels of expression, fold change and genomic context (Fig. 2B: Pearson R = 0.995, p < 0.001, R^2^ = 0.9899).

**Figure 3:**
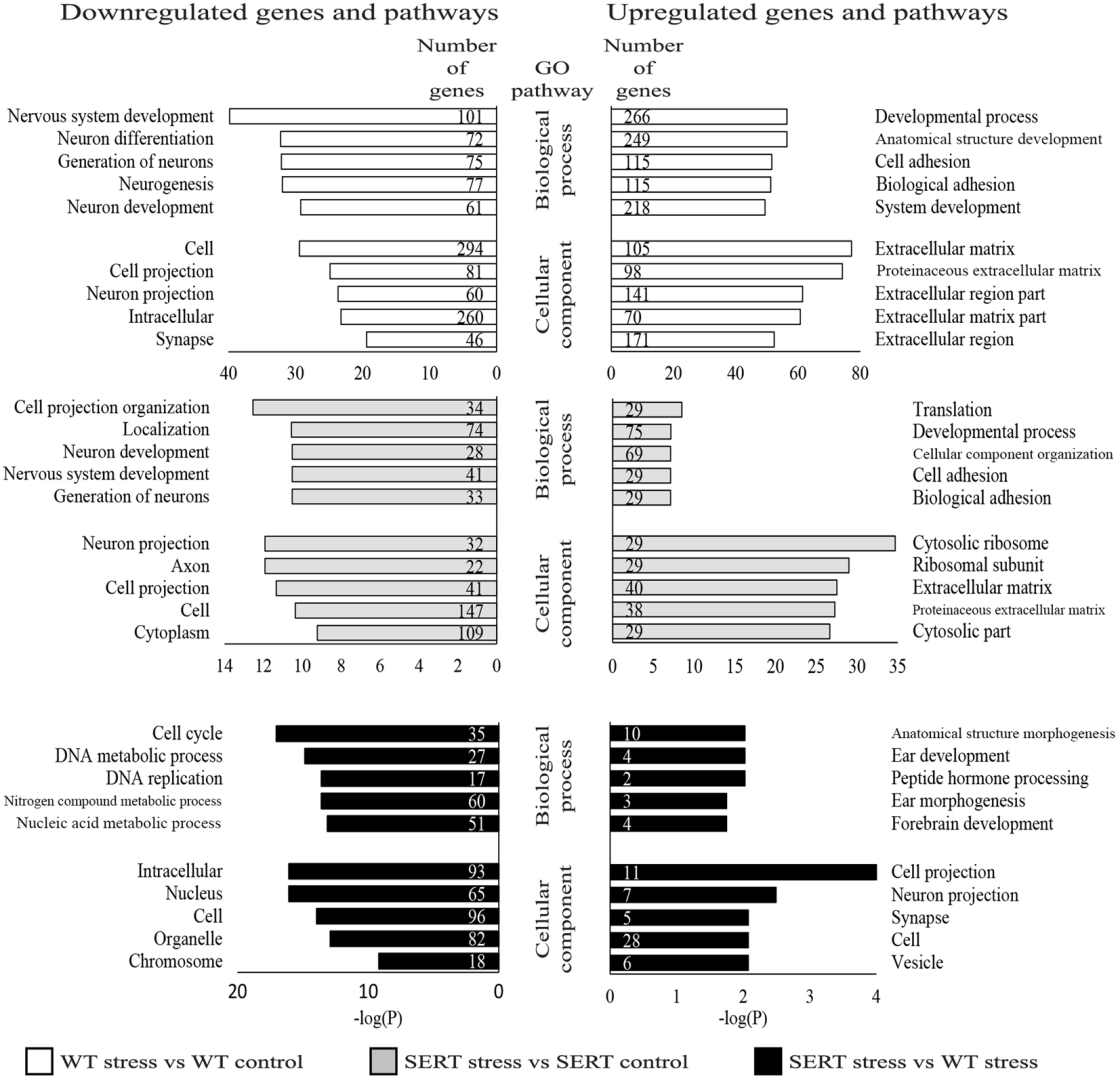
Analysis of gene ontology for biological processes and cellular component for downregulated (left panel) and upregulated (right panel) genes from WT embryos in response to stress (top panel), SERT embryos in response to stress (middle panel), and the gene x environment interaction (bottom panel).

DEG lists were submitted to a GO pathway mapping algorithm which demonstrated that many overrepresented pathways were similar in the WT and SERT embryos in response to stress (Fig. 3). Generally, both groups downregulated genes involved in neuron projection and differentiation and upregulated genes involved in extracellular matrix and adhesion; however, the number of affected genes in these pathways are much higher in the WT response than the SERT response to stress. Overrepresentation of ribosome pathways occurs only in the SERT response to stress.

To qualify communication between mother and embryo via differential gene expression in the placenta, the placenta transcriptomes were sequenced. There was no clustering of related samples due to genotype or environmental stress (Supplementary Fig. S2) and consequently, few genes were differentially expressed due to genotype or environmental conditions. In the absence of stress, only one gene (*Xaf1*) was differentially expressed in the SERT placenta compared with the WT placenta (fold change = 2.19, p = 1.06E-5).

### miRNA profiling

Comparison between miRNA expression in the SERT control versus WT control yielded no DE miRNA. When mothers were exposed to the stress paradigm, WT embryo demonstrated 157 DE miRNAs between control and stress groups, only 6 were upregulated (Supplementary Table S4A). Similar comparison between the SERT embryos under stress with the SERT controls yielded no DE miRNA. The gene x environment interaction (SERT stress versus WT stress) identified two DE miRNAs (Supplementary Table S4B).

### Integration of mRNA, miRNA and methylome datasets

Selection of DMRs that overlapped with DEGs detected 52 regions in 50 genes in the WT stress embryos and 246 regions in 146 genes in the SERT stress embryos. Retaining only genes listed in the SFARI and/or AutismKB databases identified 19 DMRs in 17 different ASD genes in the WT stress group and 67 DMRs in 29 ASD genes in the SERT stress group. There were 10 DMRs in 10 WT genes and 45 DMRs in 23 SERT genes whose methylation profile correlated with the expected change in gene expression (Table 2). All 55 DMRs were hypermethylated and expression of the associated genes are down-regulated.

**Table 2:** Integration of DEGs and DMRs datasets from embryos in WT or SERT dams in response to prenatal stress identifies genes that are differentially expressed and have differential methylation.

	Genes that are differentially expressed and have differential methylation
WT	*Cadps, Chd7, Mtss1, Nbea, Nrxn2, Nrxn3, Plekha6, Slc4a8, Stox2, Vta1*
SERT	*Amph (3), Ank2 (3), Apc (2), Atp2b2 (2), Camta1 (5), Ccdc88c (2), Celf5 (2), Celsr3, Chst10 (2), Clcn3 (2), Dlg4 (2), Kif5a, Mtss1 (4), Myo16, Nmnat2, Pcdh9, Phactr1, Plxna4 (2), Rere (3), Sema5b, Slc6a1 (2), Sqle, Syn3*

I. Parenthesis indicate the number of DMRs found within and/or surrounding each gene. II. List reflects genes that are: listed in SFARI and/or AutismKB databases, differentially expressed (fold change ≥1.5 or ≤−1.5, p ≤ 0.05) and differentially methylated (p ≤ 0.05) in the WT or SERT embryos in response to prenatal stress.

**Figure 4:**
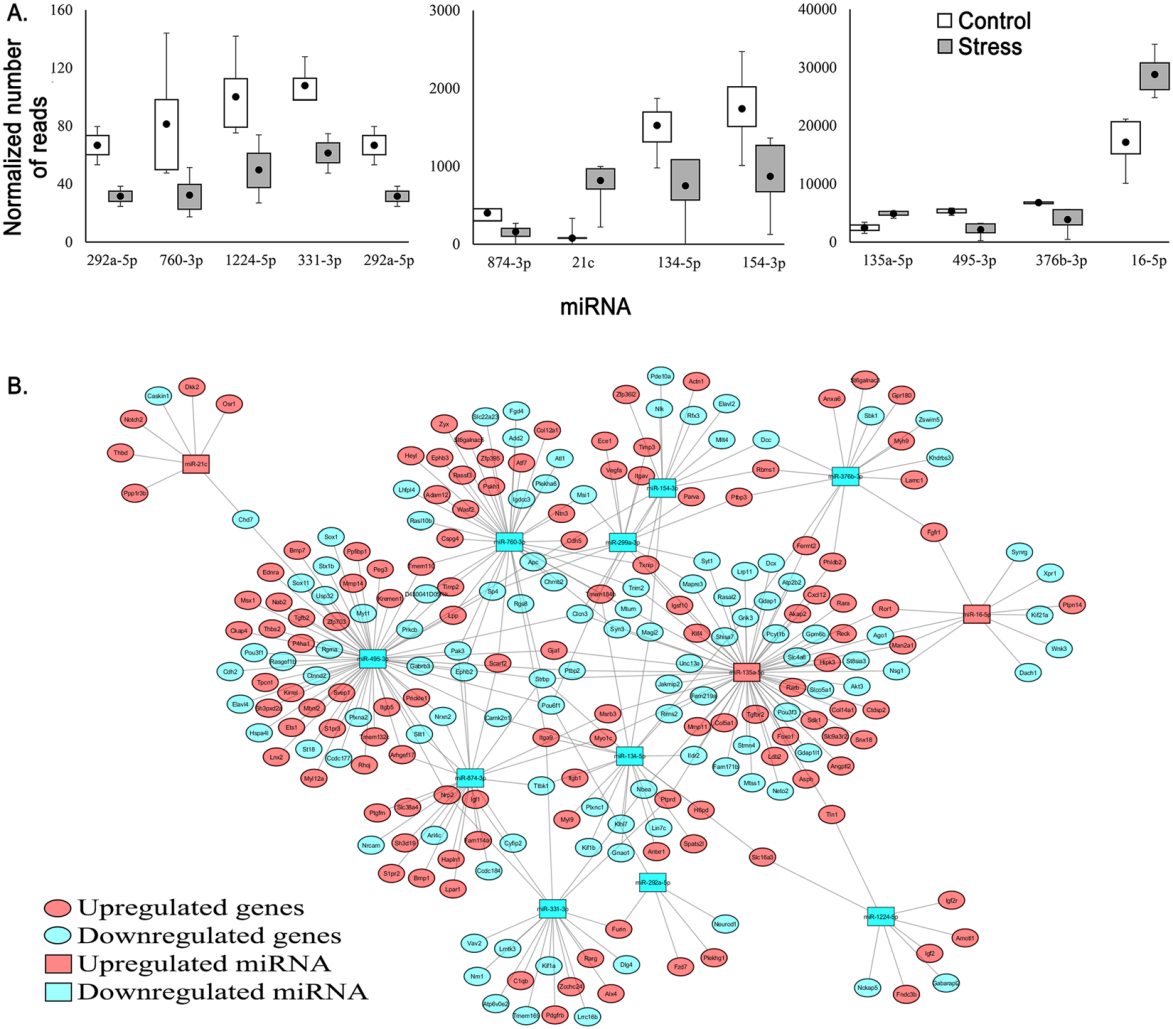
Expression level and targets of differentially expressed (DE) miRNA. (**A**) Number of reads mapping to the 13 DE miRNAs (p ≤ 0.05) that are predicted to target DE mRNA (p ≤ 0.05) in the wild-type (WT) embryos response to stress. (**B**) Cytoscape map illustrating the network of DE miRNA and their predicted DEGs.

**Figure 5:**
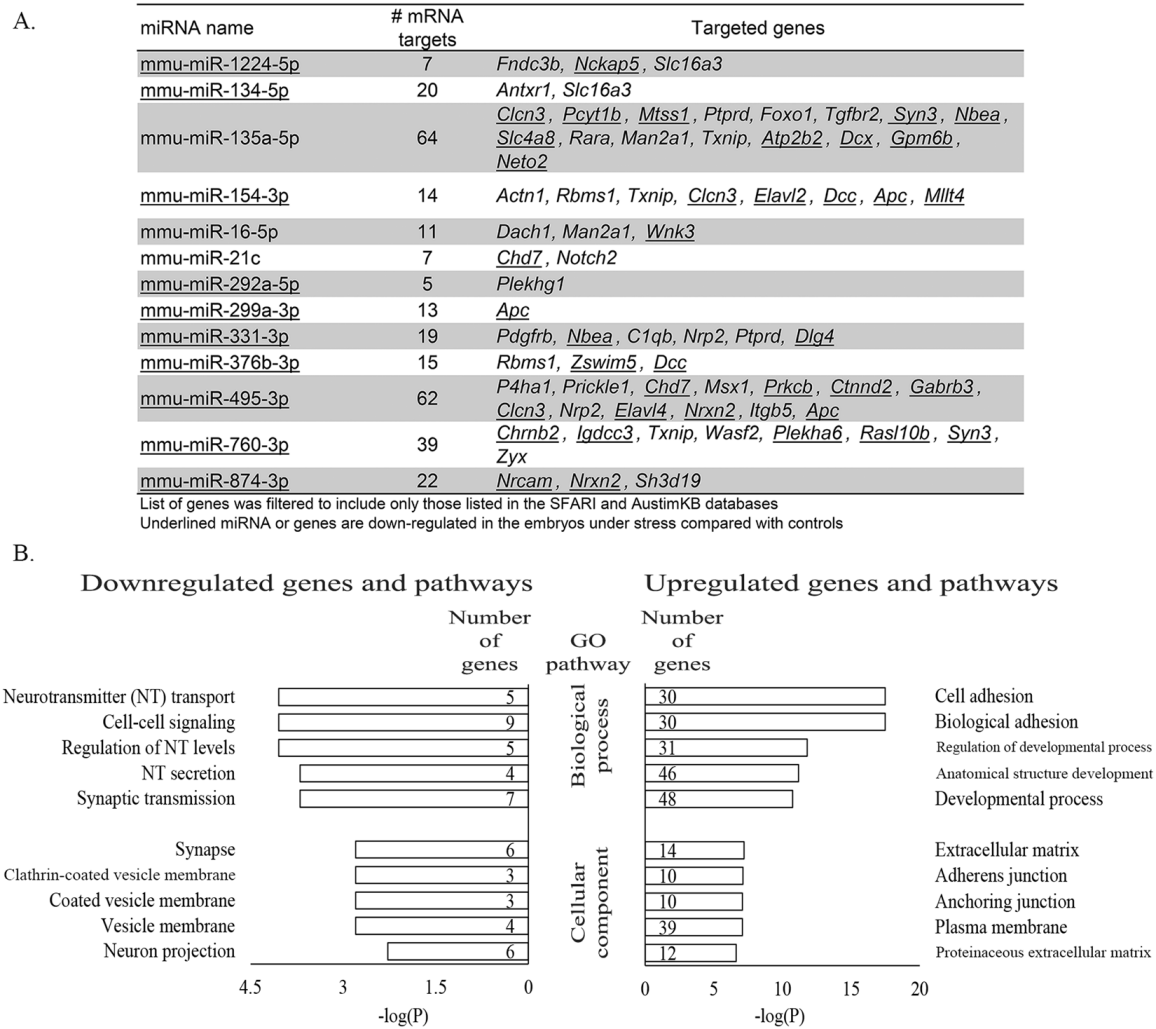
Autism spectrum disorder (ASD)-related DEGs targeted by DE miRNA. (**A**) List of 13 DE miRNA (p ≤ 0.05) and their DEGs gene targets (p ≤ 0.05) in the WT embryos in response to stress. (**B**) Analysis of gene ontology for biological process and cellular component for targeted, ASD-related DEGs for downregulated (left panel) and upregulated (right panel) genes.

Target prediction of DE miRNAs found 13 miRNAs (1224-5p, 134-5p, 135a-5p, 154-3p, 16-5p, 21c, 292a-5p, 299a-3p, 331-3p, 376b-3p, 495-3p, 760-3p, 874-3p) (Fig. 4A) that target many of the DEG in the WT stress embryos (Fig. 4B). Filtering the list of targeted DEGs to include only genes that are listed on the SFARI and/or AutismKB identified 69 genes that are targeted by the 13 DE miRNA (Fig. 5A). Pathway analysis of the 69 targeted genes show similar over- and underrepresented pathways as seen with the entire list of WT stress DEGs (Fig. 5B).

## Discussion

Animal models have been used to investigate the neurobiological, physiological, and behavioral consequences of reductions in SERT expression. Heterozygous knockout mice for the serotonin transporter gene, *Slc6a4* ^+/−^, resemble humans homozygous with the short 5′HTTLPR allele regarding SERT expression and function^32^ allowing for predictive assessments across species. These mice display a multitude of abnormalities in their physiological makeup including response to stress with elevated levels of corticosterone, adrenocorticotropic hormone, and epinephrine^32, 48, 49^ as well as in their behavioral phenotype including increased anxiety, acoustic startle, and learned helplessness^32^ which are consistent with observations made in human carriers of the short 5′ HTTLPR allele. Furthermore, the offspring of *Slc6a4* ^+/−^ mice exposed to stress demonstrate ASD-like characteristics including increased anxiety^37^ and decreased social interaction and social interest^36^, similar the increased risk of developing ASD in human children resulting from maternal polymorphism in the promoter region of the *SLC6A4* gene when combined with prenatal stress^31^.

The present study investigates mechanisms behind this phenomenon by examining epigenetic and transcriptomic profiles of mice embryo brains resulting from the interplay between maternal *Slc6a4* genotype and exposure to prenatal stress. We discuss the effect of maternal genotype in the absence of environmental stressors, the typical response to stress by embryos in *Slc6a4* ^+/+^ dams and the attenuated response to stress of embryos in *Slc6a4* ^+/−^ dams.

### Effect of maternal genotype on embryo’s brain in control environment

Comparison of the WT and SERT control embryos demonstrated that *Slc6a4* ^+/−^ maternal genotype resulted in regional hypomethylation of many regions of the genome when compared with embryos from *Slc6a4* ^+/+^ maternal genotype. Genome methylation is performed by the DNA methyltransferase enzymes (*Dnmt1, Dnmt3a* and *Dnmt3b*). In addition, the UHRF1 protein interacts with the DNMT1 enzyme at the replication fork and disruption of the DNMT1-UHRF1 interaction results in massive genomic hypomethylation^50^. Though individually the reduced expression of *Dnmt1* (−1.27 fold, p = 0.08), *Dnmt3b* (−1.33 fold, p = 0.22), and *Uhrf1* (−1.49 fold, p = 0.03) were not statistically significant, we speculate that together the reduction in expression of these 3 genes may play a role in the regional hypomethylation seen in the SERT embryos. The DMRs in the SERT control embryos did not have a direct effect on gene or miRNA expression likely due to most DMRs being in intronic and intergenic regions which are usually less significant for impacting transcription than DMRs in promoter or exonic regions. Hypomethylation plays an important role in cancer development because hypomethylation causes genome instability resulting in an increased loss of heterozygosity and oncogene activation^50, 51^. Similarly, hypomethylation of SERT embryos may have an indirect effect on the brain development though genome instability.

To qualify communication between mother and embryo via differential gene expression in the placenta, the placenta transcriptomes were sequenced. In the absence of stress, only one gene (*Xaf1*) was differentially expressed in the SERT placenta compared with the WT placenta. XAF1 has a role during implantation^52^, its aberrant expression leads to pregnancy complications^53^. and the maternal *Slc6a4* ^+/−^ genotype has been shown to affect *Xaf1* expression in the offspring in mice^37^. Offspring from *Slc6a4* ^+/−^ dams have a fifty percent chance of receiving one short 5HTTLPR allele and these embryos were removed from further analysis to minimize confounders of the maternal effect. We only found one of the six embryos (17%) collected from *Slc6a4* ^+/−^ dams with the *Slc6a4* ^+/−^ genotype suggesting that these embryos may have reduced viability compared with WT embryos potentially resulting from aberrant *XAF* expression in the placenta. While the interpretation is speculative, these observations warrant further study to define a role for increased expression of *Xaf1* in the placenta of *Slc6a4* ^+/−^ dams and viability of *Slc6a4* ^+/−^ mice compared to WT mice *in utero*.

### Typical embryo response to prenatal stress in *Slc6a4*^+/+^ mother

**Figure 6:**
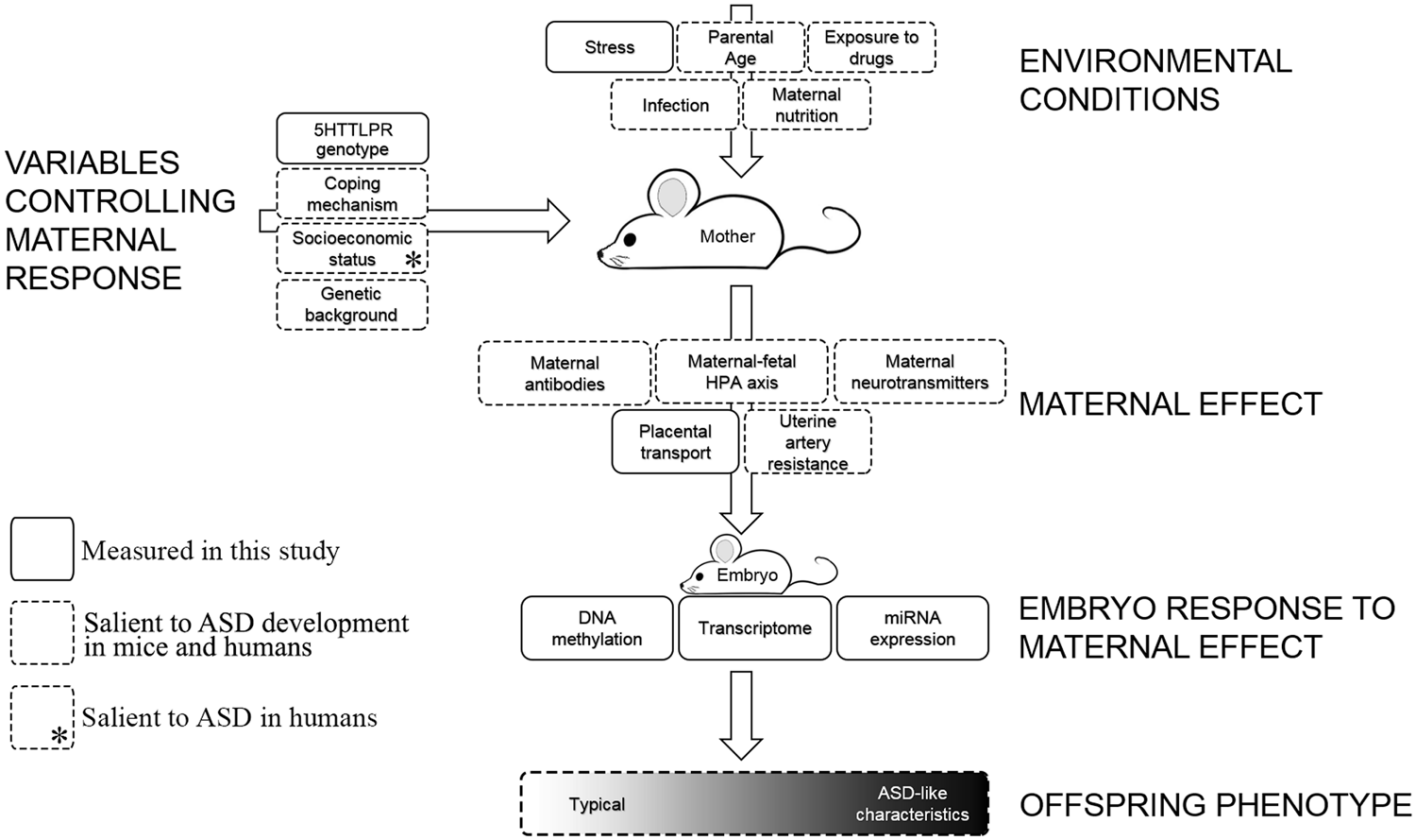
Non-genetic, *in-utero* factors that may contribute to development of neurodevelopmental disorders. A mother persistently encounters challenging environmental conditions, maternal factors impact how she relates with these conditions and impact how the stress is translated to her developing offspring. Genetically equivalent embryos face varying environmental conditions depending on the maternal environment and maternal response to her environment. Variables reported in this study using a mouse model are indicated by boxes with solid line, variables prevalent in the literature and could be tested in a mouse model and/or in humans are indicated by boxes with dotted line, and a variable that is not relevant for a mouse in laboratory conditions but salient for ASD-risk in humans is shown by an asterisk.

The typical embryo response to prenatal stress is demonstrated by WT stress embryos compared with WT control embryos. We expected that stress would have a physiological effect on the mother and the stress would be communicated to the embryo through the placenta. The placenta forms a barrier between the mother and the embryo controlling and limiting the movement of stress induced hormones^54^. In contrast to studies showing that stress caused differential expression of several genes in the placenta^55, 56^, this study did not uncover DEGs in the placenta in response to stress. Other potential maternal cues that were not considered in this report include maternal antibodies^57^, maternal-fetal HPA axis (hormones) and uterine artery resistance (blood flow)^58^ (Fig. 6). The WT embryo response to prenatal stress is quite striking as seen by dramatic changes in the methylome, transcriptome and miRNA profiles. The transcriptomic response involved 621 upregulated genes over representing pathways involved in the extracellular matrix and cell adhesion, and 388 downregulated genes associated with neuron development and projection. Other studies have also shown that prenatal stress has been associated with decreased neuron proliferation^59^ and dendrite growth^60^ in specific regions of the brain. We speculate that a coping mechanism utilized by a WT embryo when confronted with stress involves strengthening existing cellular interactions by increased expression of genes involved in cell adhesion and the extracellular matrix while simultaneously reducing genes involved in neuron growth and development that may result in erroneous connectivity.

Most DMRs found in the WT stress embryos compared with WT control embryo were hypermethylated and many located in gene exonic and regulatory regions, indicating that they are involved in controlling gene transcription. Likewise, other studies have reported an increase in methylation due to prenatal stress in targeted region of the genome including the glucocorticoid receptor gene (*Nr3c1)* ^61^ and the brain-derived neurotrophic factor (*Bdnf*)^62^. The mechanism behind the hypermethylation is unknown because there was no change in *Dnmt1, Dnmt3a*, or *Dnmt3b* expression. The list of ASD gene related DMRs include *Cadps, Chd7, Mtss1, Nbea, Nrxn2, Nrxn3, Plekha6, Slc4a8, Stox2*, and *Vta1*, where each DMR and DEG interaction is characterised by hypermethylation and decreased gene expression. Chromodomain Helicase DNA Binding Protein 7 (CHD7) is a protein involved in helicase activity and chromatin modeling. Mutations in the *CHD7* gene are responsible for the development of most cases of CHARGE syndrome^63^, an ASD syndromic subtype^64^. The products of the neurexin genes (*Nrxn2* and *Nrxn3*) function as cell adhesion molecules in the formation of neuronal synapses and are important for the development of ASD in mice^65^ and humans^66^.

The WT response to stress also involves post-transcriptional gene regulation by miRNAs. miR-21c is one of 3 miRNAs upregulated in the WT stress embryos and one of its predicted targets is *Chd7*. For *Chd7*, the two epigenetic mechanisms, hypermethylation of the genomic *Chd7* gene and increased expression of miR-21c, work together to reduce gene expression. For other gene targets, like *Nrxn2*, hypermethylation and decreased miR-874-3p expression appear to be working against each other. A single miRNA can target 200 transcripts and multiple miRNA can act upon a single transcript^67^, so interactions involving methylation status, miRNA expression and gene expression are complex. There are reports describing miRNA that are dysregulated in response to stress^68^ and in individuals with ASD^69^, but there is little overlap between these studies indicating that the role of miRNA in ASD requires more investigation^69^.

### Atypical embryo response to prenatal stress in *Slc6a4*^+/−^ mother

The SERT embryos responded to stress by significant increase in genome methylation and reduced transcriptional response illustrated by the decreased number of DEGs and absence of DE miRNA. The extensive genome-wide hypermethylation may be a result of genome instability associated with hypomethylation in the SERT control, and resulted in silencing of genes involved in the “coping” mechanism described in the WT response. Many of the DEGs and affected pathways are similar in the SERT and WT stress responses, however the number of DEGs associated with each shared pathway was always less in SERT embryos, indicating the coping mechanism may be attenuated in the SERT embryos responding to prenatal stress. Interestingly, one affected pathway unique to the SERT embryos is the ribosome pathway, with 30 genes being overexpressed only in the SERT stress embryos. Overexpression of ribosomal RNA and proteins has been described in cancers^70, 71^, though their roles beyond protein synthesis is not well understood. There is little research connecting overexpression of ribosomal protein and developmental disorders, but recently Smagin *et al*.^72^ described how chronic social defeat stress leads to development of anxiety and depression in male mice and is accompanied by upregulation of many ribosomal genes in the hypothalamus.

### Limitations

We found variation within the SERT embryo group, specifically one SERT embryo that appeared to respond to stress comparable to the WT embryo, reducing the significance of the gene vs environment comparison. We propose that this embryo may be receiving different cues from the mother^73^ and despite the combined risk of maternal genotype and prenatal stress, that this embryo is developing typically. Second, the size of a E13.5 embryo made brain dissection from the head difficult, and some trends presented may represent tissue surrounding the brain. Third, this small study presents a snapshot of the effect of maternal genotype and prenatal stress on embryonic development. A larger sample size with multiple timepoints during embryo development would provide a timeline demonstrating how embryos cope with acute or chronic stress.

## Conclusion

Based on the results and trends of this study, we propose a generalized epigenetic mechanism behind the development of ASD-like characteristics in embryos developing in *Slc6a4* ^+/−^ dams exposed to prenatal stress. Since all the embryos were genetically equivalent, all embryonic changes that occur are likely a result of maternal effect stemming from maternal *SERT* genotype and maternal stress (Fig. 6). Initially, embryos in *Slc6a4* ^+/−^ mice demonstrated regional hypomethylation compared to the WT mice, which we speculate may be due to reduced expression of embryonic methyltransferases and their related machinery^74^. The DMRs do not have a direct effect on expression of miRNA or mRNA in the embryo; however, we speculate that the regional hypomethylation may act indirectly in contributing to reduced embryo resilience to maternal effect as seen by the attenuated response to prenatal stress. The typical embryonic response to stress (i.e. embryo with a WT mother) is large and organized, involving increased methylation in transcriptionally relevant regions of many genes and differential expression of many mRNA and miRNA. This response may constitute a coping mechanism allowing for correct neural development and differentiation during adverse conditions. On the other hand, the response of an embryo with a *Slc6a4* ^+/−^ mother is diminished or sluggish; they have significant increase in genome methylation and significantly reduced transcriptomic and miRNA response. Jones *et al*. reported that embryos in the same maternal environment described in this study develop into mice displaying ASD-like characteristics^36^. We speculate that the transcriptomic and epigenetic changes reported in this study, specifically altered gene expression involved in neuron development, may effect typical brain development contributing to the development of ASD-like characteristics described by Jones *et al*.^36^. This study highlights the importance of gene/environment interactions, the unresolved role of maternal effect on embryo development, and generally contributes to our understanding of the intricacy underlying the development of complex disorders like ASD.

## Electronic supplementary material

Supplementary Information

Supplementary Table S2

Supplementary Table S3

Supplementary Table S4
