# Supplementary material for: Interplay between maternal Slc6a4 mutation and prenatal stress: a possible mechanism for autistic behavior development
Source: Sci Rep. 2017 Aug 18;7:8735. doi: 10.1038/s41598-017-07405-3 (PMC5562880; doi:10.1038/s41598-017-07405-3)
Supplement: Supplementary file 1 — Supplementary Information [file 41598_2017_7405_MOESM1_ESM.pdf]

Summary of Supplementary Information for

Title: Interplay between maternal *Slc6a4* mutation and prenatal stress: a possible mechanism for autistic behaviour development

Calvin P. Sjaarda PhD[1,2], Patrick Hecht PhD [3], Amy J.M. McNaughton PhD [1,2], Audrina Zhou[1,2], Melissa L. Hudson BSc[1,2], Matt J. Will PhD[4], Garth Smith MBBS FRCPC[5,6], Muhammad Ayub MD, MRCPsych[1], Ping Liang PhD [7], Nansheng Chen PhD [8], David Beversdorf MD[3,9], and Xudong Liu PhD[1,2*]

[1] Department of Psychiatry, Queen's University, Kingston, Ontario, Canada

[2] Queen's Genomics Lab at Ongwanada (QGLO), Ongwanada Resource Center, Kingston, Ontario, Canada

[3] Interdisciplinary Neuroscience Program, University of Missouri, Columbia, Missouri, USA

[4] Psychological Sciences and Bond Life Sciences Center, University of Missouri, Columbia, Missouri, USA

[5] Department of Pediatrics, Queen's University, Kingston, Ontario, Canada

[6] Child Development Centre, Hotel Dieu Hospital, Kingston, Ontario, Canada

[7] Department of Biological Sciences, Brock University, St. Catharines, Ontario, Canada

[8] Department of Molecular Biology and Biochemistry, Simon Fraser University, Burnaby, British Columbia, Canada

[9] Departments of Radiology, Neurology, and Psychological Sciences, and the Thompson Center for Autism and Neurodevelopmental Disorders, and William and Nancy Thompson Endowed Chair in Radiology, University of Missouri, Columbia, Missouri, USA

List of Figures:

Supplementary Figure S1: Quantification of the ERCC RNA Spike-In Control Mixes to assess the dynamic range, lower limit of detection and fold-change response of the RNA-Seq methodology.

Supplementary Figure S2: Heatmap portraying the transcriptome of the placenta samples demonstrates little clustering of related genotypes or environmental conditions.

List of Tables:

Supplementary Table S1: qPCR primers designed in NCBI's primer blast to validate the RNA-seq expression level of 12 genes

Supplementary Table S2: Differentially methylated regions in A: SERT control embryos compared with WT control embryos, B: WT embryos in response to stress, C: SERT embryos in response to stress (separate Excel file).

Supplementary Table S3: Differentially expressed genes in A: WT embryos in response to stress, B: SERT embryos in response to stress and C: gene x environment interaction (SERT stress versus WT stress) (separate Excel file).

Supplementary Table S4: Differentially expressed miRNA in A: WT embryos in response to stress and B: gene x environment interaction (SERT stress versus WT stress) (separate Excel file).

Supplementary Materials and Methods

Animal model. Ten *Slc6a4*$^{-/-}$ male mice were bred with 20 wild-type (WT: *Slc6a4*$^{+/+}$) female mice on a C57BL/6J background (Jackson Laboratories, Bar Harbor, ME). The resulting WT female offspring underwent another round of breeding with male *Slc6a4*$^{+/-}$ mice to generate the experimental dams and sires used in this study. Through these initial two rounds of breeding, only WT females were used in breeding to prevent any residual effects of intrauterine environment or abnormal maternal care on the offspring. All mice were housed in 7 x 11.5 x 4.75 in^3 Plexiglas cages with Aspen bedding and a cotton nestlet and had *ad libitum* access to food and water. The rodent diet used in this study started with the AIN-93G purified diet profile (Dyets Inc., #110700, Bethlehem, PA) as the base with added corn and soy oil (2:1 ratio). Males and females were housed separately with littermates in groups of 1-4 per cage. Animals were kept in rooms with a temperature of 21 °C and a 12:12 light: dark cycle with lights on at 7:00 AM.

Experimental Breeding. Experimental dams (*Slc6a4*$^{+/+}$ and *Slc6a4*$^{+/-}$) were housed in pairs of two and bred with WT males. To increase the likelihood of copulation and pregnancy, estrous cycle was examined via visual inspection of the vagina as previously described [1]. Females in proestrus or estrus were paired with the male and inspected for a vaginal plug the following morning. Identification of a plug was marked embryonic day 0.5 (E0.5) and the females were then housed singly throughout the duration of the pregnancy.

Prenatal Stress. Pregnant mice randomly selected using Microsoft Excel's RANDBETWEEN function to be included in the stress condition were exposed to restraint stress on E12.5. The restraint stress consisted of pregnant mice being transferred into the testing room and placed in a clear Plexiglas tube (internal diameter = 1.33 inches; length = 5 inches) lasting for 2 hours.

Acute prenatal stress at E12.5 has been shown in previous studies to produce detrimental effects in typical neurodevelopmental processes resulting in altered social and anxiety-like behaviour in the offspring [2, 3]. Restraint stress was used as the acute stressor in this study due to its common use in animal models of stress and its well defined effects on the hypothalamic-pituitary-adrenal (HPA) stress pathway [4]. We also see value in expanding this study across different stages of gestation, and the use of a single stressor allows for consistent assessment of the effect of this stress over time. Exposure to 2 hours of restraint stress has been shown to produce significant effects on HPA activity, gene expression, and behavioral activity [5-7]. Following the restraint stress period, mice were placed back into their home cage and returned to the colony room.

Tissue Collection. Embryonic tissue was collected at E13.5 by a male experimenter [8] (Table 1). For embryonic brain tissues, the experimental dams were first euthanized and had the abdominal cavity opened promptly. The uterine horn containing the embryos was removed and placed in ice cold PBS. Individual embryos were then removed and rapidly decapitated. Embryo heads were collected in separate microcentrofuge tubes, immediately frozen in liquid nitrogen and stored in -80°C until further processing.

Nucleic acid isolation and library construction. 10 mg of tissue was minced in a microcentrifuge tube using a disposable pestle and DNA was extracted using the PrepEase Genomic DNA Isolation Kit (Affymetrix, Santa Clara, CA). DNA concentration and purity was measured by spectrophotometer and visualized by gel electrophoresis. Three µg of DNA was fragmented using a Covaris S2 (Covaris, Woburn, MA) with the 200 base pairs DNA manufacturer's protocol – frequency sweeping mode with one cycle, bath temperature limit of 8°C, total processing time of 3 min consisting of three 60 sec treatments of 10% duty cycle, intensity of 5, and 200 cycles per burst. Following fragmentation, samples were purified with

SPRI-Select beads (Beckman Coulter, Mississauga, ON) using a 1:1.9 DNA volume to beads volume ratio and following manufacturer's protocol. Fragmentation quality was assessed using agarose gel electrophoresis and samples were quantified using Qubit dsDNA BR Assay kit with Qubit Fluorometer 2.0 (ThermoFisher Scientific, Carlsbad, CA). Methylated DNA was captured from 600 ng purified, fragmented DNA using MethylMiner Methylation DNA Enrichment (Invitrogen, Carlsbad, CA), eluted using high-salt buffer (2000 mM NaCl), precipitated with ethanol and resuspended in 25 µL 0.1X TE. Fragmented DNA ends were repaired using the NEB Next End Repair Module (New England BioLabs Inc., Ipswich, MA) following the manufacturer's protocol using half-reaction volumes. Samples were purified using a 1:1.9 DNA: beads ratio with SPRI-Select beads and eluted in 30 µL 0.1x TE. Barcoded adaptors were ligated to end-repaired DNA using the Ion Xpress Plus gDNA Fragment Library Kit and Ion Xpress Barcoded Adaptors (ThermoFisher Scientific). Following ligation, samples were purified as described following end-repair and the adaptor-ligated libraries were amplified with the Ion Xpress Plus gDNA Fragment Library kit (ThermoFisher Scientific), following the 50-100 ng input manufacturer's protocol. Size selection of the amplified libraries targeted 100-250 bp fragments using SPRI-Select beads (Beckman Coulter) in a two-step protocol, 1:0.85-0.9 (DNA: beads) ratio followed by 1:0.2-0.25 (DNA: beads) ratio. After elution in 30 µL 0.1X TE, the size-selected libraries were quantified using the Ion Library Quantification Kit (ThermoFisher Scientific) using half-reaction volumes on an Applied BioSystems ViiA 7 (ThermoFisher Scientific) and size distribution was assessed using the Bioanalyzer 2100 (Agilent Technologies).

Another 10 mg of tissue was minced using a disposable pestle, homogenized and total RNA was recovered using the PureLinks RNA Mini Kit (ThermoFisher Scientific). Residual genomic DNA was removed on the column with the PureLinks DNase Set (ThermoFisher Scientific) and

RNA samples were randomly spiked with either ERCC ExFold RNA Spike In Mix 1 or Mix 2 (ThermoFisher Scientific). Total RNA concentration and integrity were determined using the Qubit Fluorometer 2.0 (ThermoFisher Scientific) and the Bioanalyzer 2100 (Agilent Technologies). mRNA was isolated from 10 ug of total RNA using oligoDT-coated beads from the Dynabeads mRNA Direct micro kit (ThermoFisher Scientific) following the recommended protocol for high input of total RNA. Libraries were constructed using the Ion Total RNA-Seq Kit v2 for whole transcriptome libraries (ThermoFisher Scientific). Complimentary DNA was amplified by PCR using the IonXpress RNA 3' Barcode Primer to allow for multiplex sequencing. Library yield and size distribution was assessed using the Bioanalyzer 2100 (Agilent Technologies).

To isolate miRNA, 10 mg of tissue was minced using a disposable pestle homogenized and small RNA was isolated using the *mir*Vana miRNA Isolation Kit (ThermoFisher Scientific) following the enrichment procedure for small RNAs. Small RNA concentration and integrity were measured using the Qubit Fluorometer 2.0 (ThermoFisher Scientific) and the Bioanalyzer 2100 (Agilent Technologies). Library construction was performed using the Ion Total RNA-Seq Kit v2 for small RNA libraries (ThermoFisher Scientific) using the IonXpress RNA 3' Barcode Primer to allow for multiplex sequencing. Library yield and size distribution was assessed using the Bioanalyzer 2100.

Next generation sequencing. Methylome, transcriptome and miRNA libraries were diluted to 80, 90 and 100 pM respectively prior to templating and sequencing. Templating was performed using the Ion PI Hi-Q OT2 200 Kit with the Ion OneTouch 2 System (ThermoFisher Scientific). Sequencing was performed using the Ion PI Hi-Q Sequencing 200 Kit, on a PI v3 chip, with the Ion Proton System (ThermoFisher Scientific). For the methylated DNA libraries, the Generic

Sequencing run plan was used with 600 flows. The sequence was aligned using the Torrent Mapping Alignment Program (TMAP) to the mm10 reference genome in the Ion Torrent Suite Software on the Torrent Server to generate aligned bam files with 19.40 ± 1.03 million mapped reads for each sample. The mRNA libraries were sequenced using the Ion RNA Whole Transcriptome run plan with 560 flows was used to generate 18.86 ± 2.10 million reads for each sample. The file exporter plugin in the Ion Torrent Suite Software on the Torrent Server (ThermoFisher Scientific) generated fastq files which were used for downstream analysis. The miRNA libraries were sequenced with the Ion RNA small run plan with 520 flows to generate 4.77 ± 0.13 million reads per sample. The file exporter plugin was used to export fastq files for analysis.

Tissue dissection, DNA and RNA extraction, library preparation and sequencing were performed blind to all metadata information about the samples. Data analysis was not performed blind to metadata information about the samples.

Bioinformatic analysis. DNA methylation was assessed using R-based pipelines MEDIPS [9] and MethylAction [10]. The MEDIPS pipeline (parameters: uniq=1e^{-3}, extend=300, shift=0, ws=100) used the edgeR test to generate a list of differentially methylated regions (DMRs) that was filtered based on p-value <0.05, minRowSum=10 and false discovery rate (FDR) = 0.1. One sample with low enrichment of methylated DNA (WT control, E23L1) was removed from further methylome analysis. The preprocessing of the bam files using MethylAction was performed using a fragment size of 120bp and a window size of 100bp, followed by DMR detection using the recommended defaults.

The transcriptome fastq files were imported into Partek Flow version 5.0.16.1113 and analyzed with the WT pipeline for Ion Torrent workflow. Briefly, samples were preprocessed by trimming

adapters and trimming reads before a two-step alignment using TopHat2 [11] and Bowtie2 [12]. Unaligned reads were analyzed for QA/QC on ERCC controls to assess the dynamic range, lower limit of detection and fold-change response as described in the ERCC RNA Spike-In Control Mixes User Guide (Thermofisher Scientific) (Supplementary Fig. S1). The aligned reads were quantified to the RefSeq Transcripts 2016-0-02 annotation model by Partek E/M to generate gene and transcript counts. The gene counts were normalized by the total count plus 1.0E-4 normalization, and differential gene expression was determined using the gene specific analysis (GSA) algorithm. Differentially expressed genes were filtered based on p-value ≤0.05, fold change >1.5 or <-1.5 and average coverage of ≥15 normalized reads. A subset of differentially expressed genes were validated by qPCR.

The miRNA fastq files were imported into Partek Flow and analyzed with the small RNA pipeline for Ion Torrent. Reads were aligned with Bowtie2 [12] and quantified to the miRBase mature microRNAs v21 annotation model. The miRNA counts were normalized using total count plus 1.0E-4 normalization and differentially expressed miRNA were determined using the GSA tool. Differentially expressed miRNAs were filtered based on p-value ≤0.05, fold change >1.5 or <-1.5 and average coverage of ≥15 normalized reads.

Validation of gene expression. To validate the RNA-seq data, 12 DEGs were selected that reflected various levels of expression, fold change and genomic context, and their relative mRNA levels was determined using quantitative real-time PCR (qPCR). 40 ng of mRNA from each sample was reverse transcribed using the SuperScript IV First-Strand Synthesis kit (Invitrogen) in an Applied Biosystems GeneAmp PCR System 9700 (ThermoFisher Scientific). The cDNA was amplified in the ViiA7 Real-Time PCR machine (ThermoFisher Scientific) with a two-step PCR protocol (95℃ for 10 m followed by 40 cycles of 95℃ for 15 s and 60℃ for 1

m) using the Power SYBR Green PCR Master Mix (ThermoFisher Scientific) and primers (Sigma-Aldrich, Oakville, ON) designed with NCBI's primer blast (Supplementary Table S1) employing the sample maximization method to minimize technical variation [13]. Each reaction was performed in triplicate and dissociation curves were generated for all reactions to ensure primer specificity. All target genes were normalized to three reference genes (*Sdha*, *Actb* and *Pgk1*) and the relative quantification using determined using the comparative Ct method.

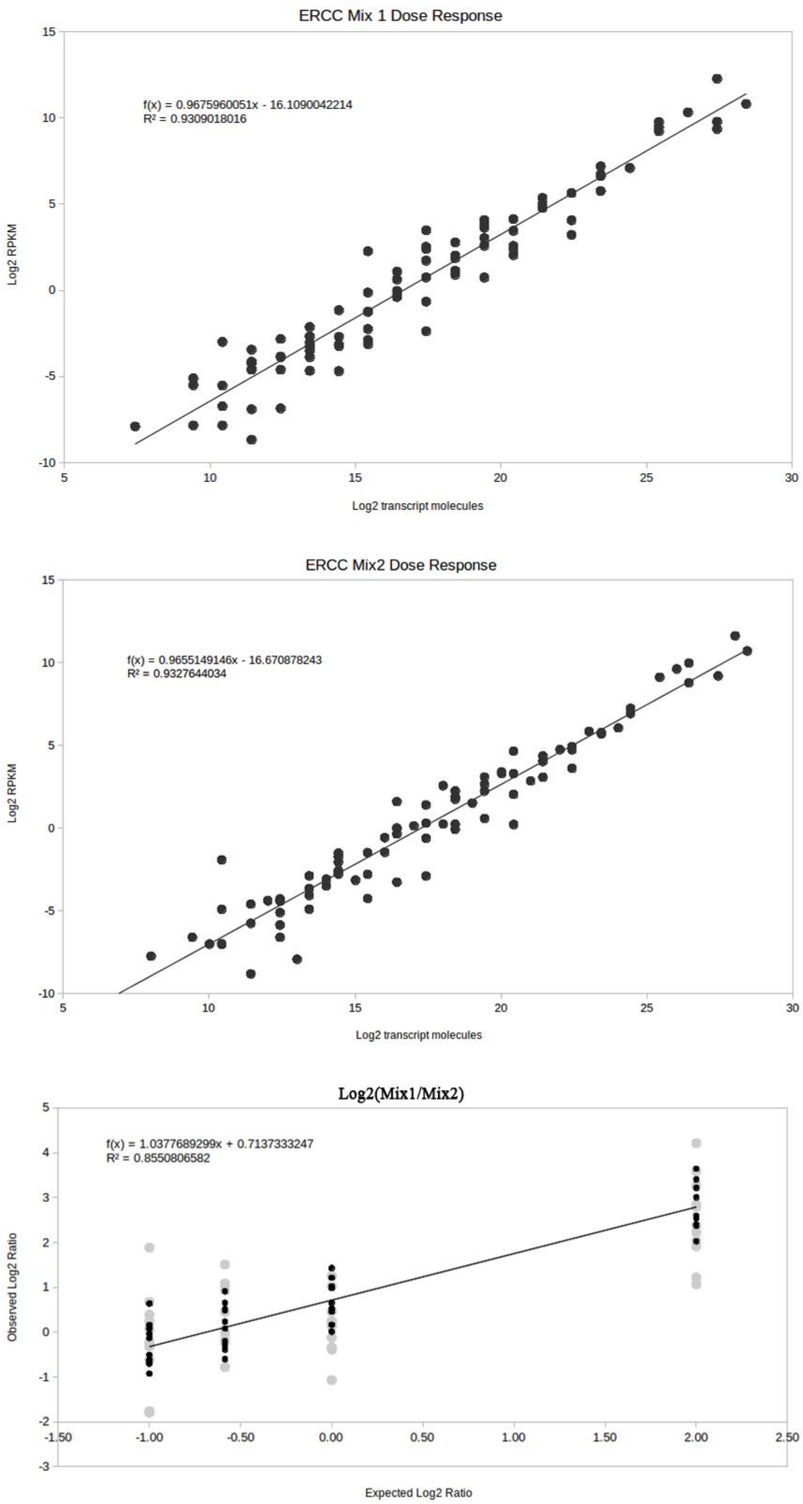

.

Supplementary Figure S1: Quantification of the ERCC RNA Spike-In Control Mixes to assess the dynamic range, lower limit of detection and fold-change response of the RNA-Seq methodology.

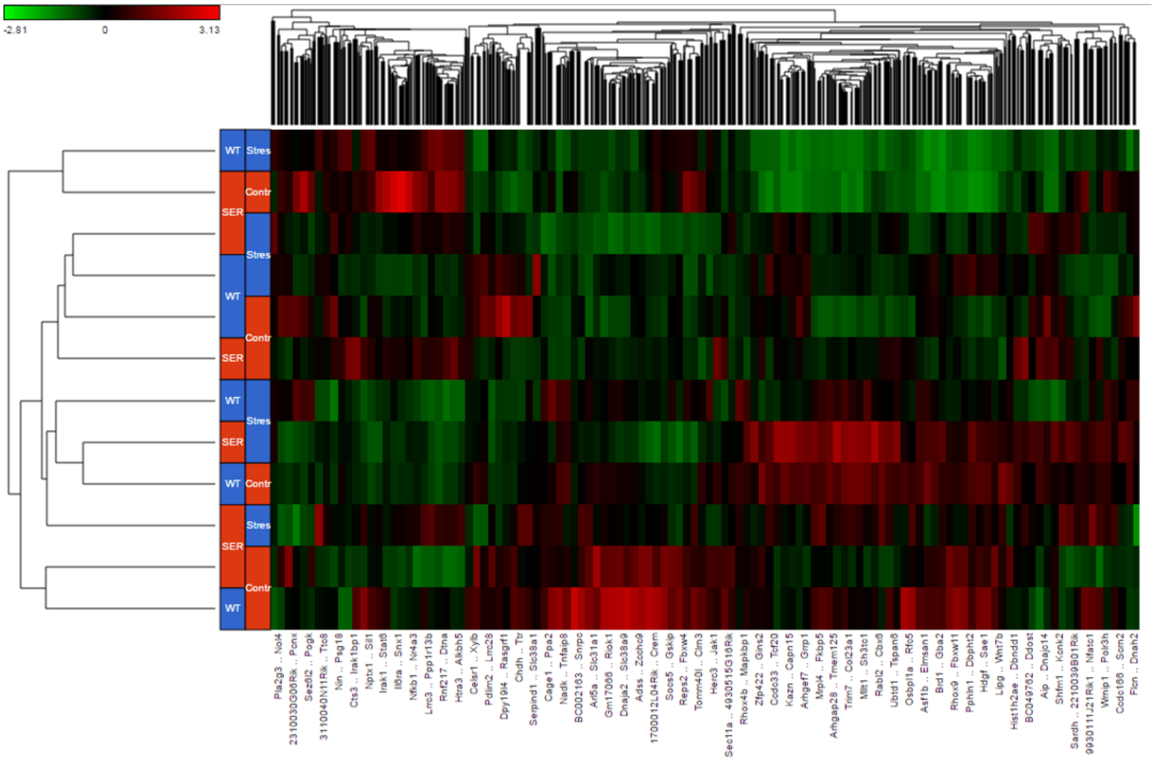

Supplementary Figure S2: Heatmap showing that the transcriptome of the placenta samples demonstrated little clustering of related genotypes or environmental conditions.

Supplementary Table S1: qPCR primers designed in NCBI's primer blast to validate the RNA-seq expression level of 12 genes

Gene name	Ref-seq ID	Gene location	Forward primer (5' - 3')	Reverse primer (5' - 3')
Nrxn2	NM_001205234.1	chr19:6428049-6532148	GGCTGAGTGTCCAAGTGATGA	AGTCCTCCGTGATAATGGGC
Chd7	NM_001277149.1	chr4:8751505-8866655	ATCCAACACCTCGCAGTCAG	CACTAGGCGGCTGCTCATAA
Nrp2	NM_001077403.1	chr1:62704205-62815637	ACCCATATCAGCTTTTGCAGGT	TCTCATCTTCATAGCCCTCTCC
Lamb1	NM_008482.2	chr12:31265421-31329493	GAAGCCGAGGAAGCCAATCT	AATAAGCCCCTTCAGGCACC
Apc	NM_007462.3	chr18:34261023-34318581	GTTTTGAGTTCTAGCGGCACG	ACCAAGCATTGACAACAAGGA
Cacna1g	NM_009783.3	chr11:94409145-94473617	CCTGGAGACACAGAGTACGG	AGGCTCCACTGTCTGCCTT
Capn6	NM_007603.3	chrX:143803644-143820460	CATTTTTGACACGCAGGCCA	CATCGAGAGTAACCTGCCCC
Dcc	NM_007831.3	chr18:71258990-72350446	CAACAATGCCGGAGAAGGTG	CCGAGGTGGGGAAATCATCAA
Igf2	NM_001315488.1	chr7:142653816-142655810	CGCTTCAGTTTGTCTGTTCGG	AGCAGCACTCTTCCACGATG
Celf5	NM_176954.3	chr10:81462326-81482605	CTTCCTCACATACTGCGCCA	TTCACTGTCTGCCGGTTTCA
Dlg4	NM_007864.3	chr11:70018920-70044695	AGATGAAGACACGCCCCCTC	CCTGCAACTCATATCCTGGGG
Slc1a2	NM_001077514.3	chr2:102659285-102781534	GCGACAAGCTGGGGAAAAAT	GACCACATCAGGGTGGATGG
Pgk1	NM_008828.3	chrX:106187251-106203279	GTCGTGATGAGGGTGGACTT	AACGGACTTGGCTCCATTGT
Sdha	NM_023281.1	chr13:74323087-74350208	AAGAAGCCGTTTGGGGAACA	ACAGTCAGCCTCATTCAAGGT
Actb	NM_007393.5	chr5:142903798-142905686	TTCTACAATGAGCTGCGTGTG	GGGGTGTTGAAGGTCTCAAA

Reference List

1. Byers,S.L., Wiles,M.V., Dunn,S.L., & Taft,R.A. Mouse estrous cycle identification tool and images. *PLoS. One.* **7**, e35538 (2012).

2. Stevens,H.E., Su,T., Yanagawa,Y., & Vaccarino,F.M. Prenatal stress delays inhibitory neuron progenitor migration in the developing neocortex. *Psychoneuroendocrinology* **38**, 509-521 (2013).

3. Lussier,S.J. & Stevens,H.E. Delays in GABAergic interneuron development and behavioral inhibition after prenatal stress. *Dev. Neurobiol.* **76**, 1078-1091 (2016).

4. Buynitsky,T. & Mostofsky,D.I. Restraint stress in biobehavioral research: Recent developments. *Neurosci. Biobehav. Rev.* **33**, 1089-1098 (2009).

5. Spiers,J.G. *et al.* Noninvasive assessment of altered activity following restraint in mice using an automated physiological monitoring system. *Stress.* **20**, 59-67 (2017).

6. Zimprich,A. *et al.* A robust and reliable non-invasive test for stress responsivity in mice. *Front Behav. Neurosci.* **8**, 125 (2014).

7. Jeong,J.Y., Lee,D.H., & Kang,S.S. Effects of chronic restraint stress on body weight, food intake, and hypothalamic gene expressions in mice. *Endocrinol. Metab (Seoul.)* **28**, 288-296 (2013).

8. Sorge,R.E. *et al.* Olfactory exposure to males, including men, causes stress and related analgesia in rodents. *Nat. Methods* **11**, 629-632 (2014).

9. Lienhard,M., Grimm,C., Morkel,M., Herwig,R., & Chavez,L. MEDIPS: genome-wide differential coverage analysis of sequencing data derived from DNA enrichment experiments. *Bioinformatics.* **30**, 284-286 (2014).

10. Bhasin,J.M., Hu,B., & Ting,A.H. MethylAction: detecting differentially methylated regions that distinguish biological subtypes. *Nucleic Acids Res.* **44**, 106-116 (2016).

11. Kim,D. *et al.* TopHat2: accurate alignment of transcriptomes in the presence of insertions, deletions and gene fusions. *Genome Biol.* **14**, R36 (2013).

12. Langmead,B. & Salzberg,S.L. Fast gapped-read alignment with Bowtie 2. *Nat. Methods* **9**, 357-359 (2012).

13. Hellemans,J., Mortier,G., De,P.A., Speleman,F., & Vandesompele,J. qBase relative quantification framework and software for management and automated analysis of real-time quantitative PCR data. *Genome Biol.* **8**, R19 (2007).
